# Dentoskeletal modifications in Class II deep bite malocclusion treatment with anterior bite plane functional appliance

**DOI:** 10.4317/jced.54092

**Published:** 2017-08-01

**Authors:** Domenico Ciavarella, Michele Laurenziello, Laura Guida, Graziano Montaruli, Crescenzio Gallo, Michele Tepedino, Lorenzo Lo Muzio

**Affiliations:** 1 Department of Clinical and Experimental Medicine, University of Foggia, Foggia, Italy

## Abstract

**Background:**

A treatment modality for Class II division 1 malocclusion is discussed. Orthodontic treatment of patients with deep bite and Class II malocclusion is an important challenge in clinical practice. The aim of this work is to compare the efficacy of anterior bite plane functional appliance (ABPFA) by assessing the changes in different times with untreated patients by literature.

**Material and Methods:**

The study group comprised 22 subjects with Class II division 1 malocclusion and hypo-divergent. Eligibility criteria for this study were: dental Class II division 1 malocclusion, hypo-divergent skeletal pattern, late mixed or permanent dentition. We analyzed with the use of stable bone structure (ASCB) at two different times: pre-treatment (T0) and post-treatment (T1) after 24 months. Inter-group differences were evaluated with paired samples t-test at the *P*<0.05 level.

**Results:**

No statistical significant differences were found in cephalometric skeletal measurements, whereas dental parameters showed a significant different overjet, which was significantly reduced (6 mm at T0 vs. 5 mm at T1) in our series.

**Conclusions:**

In ABPFA group, the treatment effects were reduce mainly Class II malocclusion, overjet and overbite alteration. This appliance seems to suggest a significant beneficial effect in mandible displacement by reducing the counter clockwise rotation of the mandible, which is further confirmed by the almost absence of modifications of ArGoMe and SNGoMe angles. The ABPFA is particularly suitable to reduce the non-desirable dental effects represented by lower incisors pro inclination, and upper incisors retro-inclination.

** Key words:**Orthodontics, Functional orthodontics, Class II malocclusion, Anterior bite plane functional appliance.

## Introduction

Class II malocclusion with deep bite was described by Strang as “a condition with overlapping of the upper anterior teeth over the lowers in the vertical plane” (1). It may be caused by many conditions, i.e.: incisor superocclusion (2), mesiodistal tooth size (3), molar infraocclusion (4), vertical maxillary over growth (5). Its treatment is difficult; in addition, the therapeutic approach is quite different according to the cause of the deep bite. In case of anterior tooth extrusion a fixed appliance may be used (6), whereas for excess of vertical maxillary growth many authors suggest to use functional appliances (in children) (7) or surgical approaches (in adults) (8).

In children, using an anterior bite plane seems to be a viable orthodontic approach to the treatment of this type of malocclusion (9). In the present paper, effects of the anterior bite plane functional appliance (ABPFA) on class II malocclusion with deep bite have been investigated.

## Material and Methods

Twenty-two patients (mean age: 9.46 ± 1.60 years old) with hypo-divergent growth (HG) and deep bite class II malocclusion were selected and examined at the Section of Orthodontics, University of Foggia. The participants and their parents provided written informed consent to be involved in the study.

Inclusion criteria were: i) hypo-divergent skeletal pattern (NSL/ML ≤ 25°; ANB ≥ 3.5; overjet ≥ 4 mm; ML/SN ≤ 30°); ii) dental Class II, Division 1 occlusal relationship (defined as more than a half-cusp molar discrepancy bilaterally and an overjet greater than 4 mm); iii) late mixed or permanent dentition, and adequate growth potential. Growth potential was evaluated using the cervical-vertebral maturation method (CVM) (10). All patients were classified within CS2 and CS3 stage.

Exclusion criteria were: i) unilateral or bilateral cross bite; ii) hyper-divergent facial growth; iii) CS4 or CS5 cervical-vertebral maturation stage; iv) oral or systemic diseases; v) missing teeth; vi) congenital cranio-facial malformations; vii) previous orthodontic treatment.

**Figure 1:**
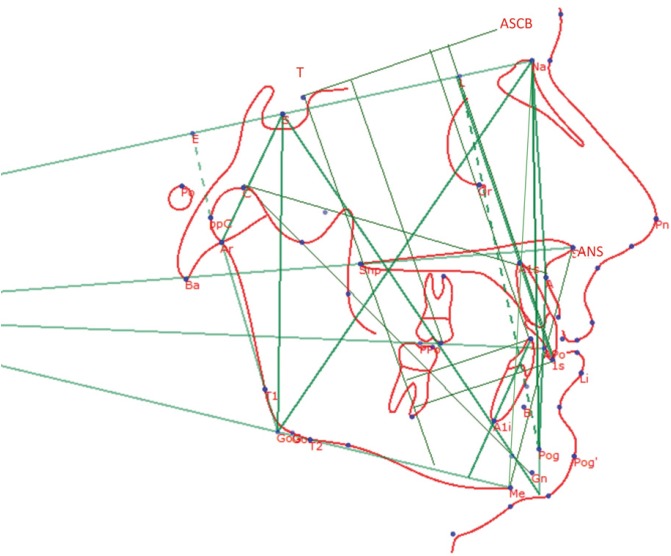
Cephalometric analysis used for the assessment of the cases treated with ABPFA.

Pre-treatment and post-treatment records included casts of maxillary and mandibular dental arches, photographs, panoramic radiographs and lateral-head films. Head films were taken with the patient fixed in a cephalostat in centric occlusion, with adequate visualization of reference structures, and no appreciable rotation of the head. Cephalometric evaluation was performed before treatment (T0), and after 24 months from the beginning of treatment (T1). Cephalometric landmarks are shown in Figure 1 and detailed in  Table 1.

**Table 1:**
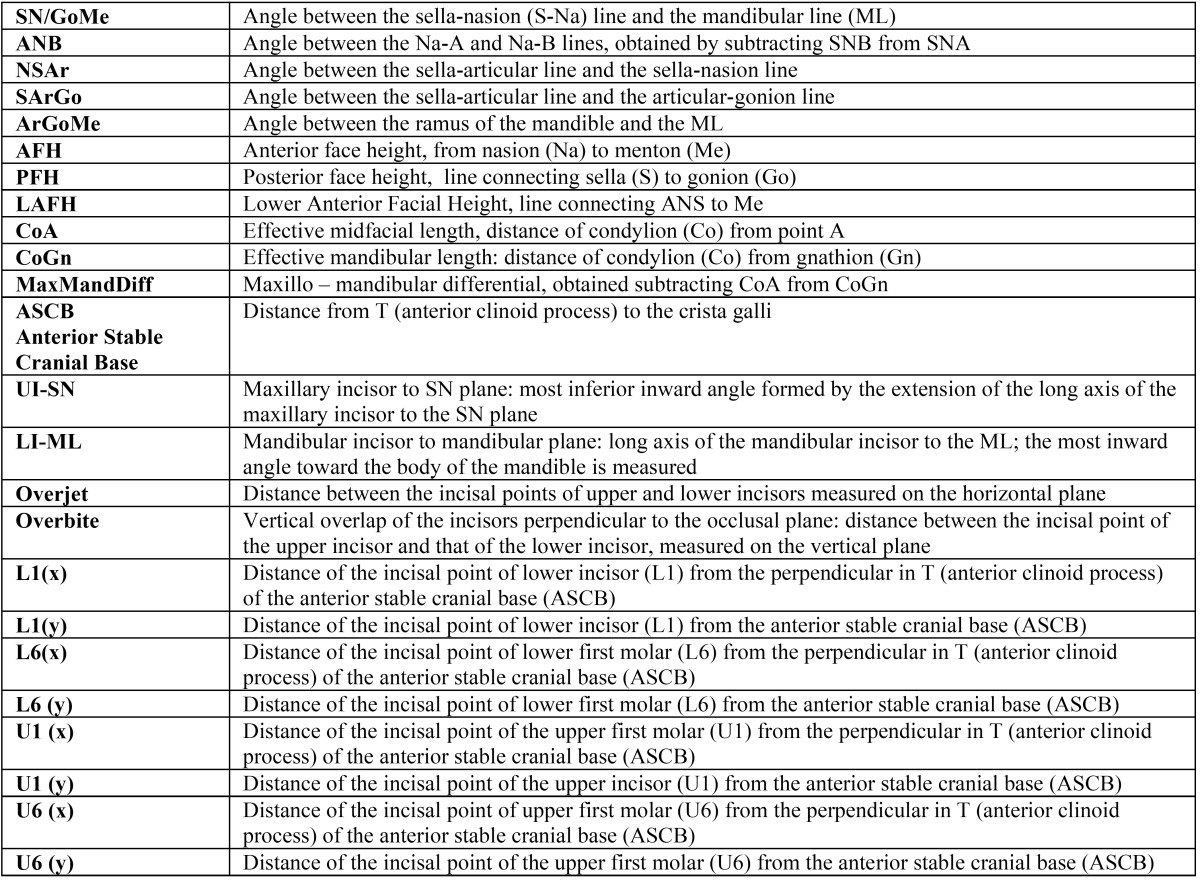
Cephalometric angular and linear skeletal/dental measurements.

The size of each patient’s radiographs were calibrated using the anterior stable cranial base (ASCB), located between the foramen caecum (F) and anterior clinoid process (T) 11. This is a stable reference structure, because the ASCB acquires its definitive dimensions between the ages of 6 and 8 years, after which the anterior cranial base develops exclusively in front of the foramen caecum (11). Cephalometric measurements were performed two times by the same examiner: for each measurement the mean of the two values was used for statistical analysis.

-Functional appliance description

**Figure 2:**
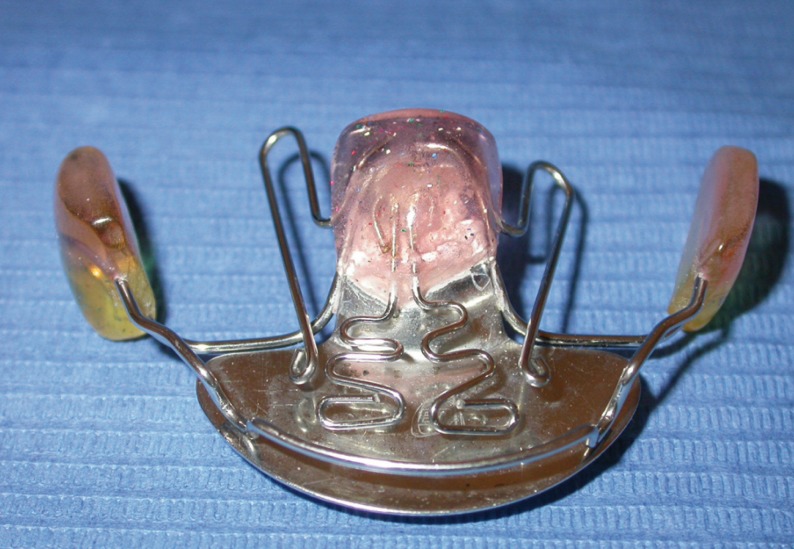
Anterior bite plane functional appliance (ABPFA).

The ABPFA was custom made for each patient by a dental technician. Acrylic components consisted of a palatal button with 2mm of clearance from the tissue, and a vestibular pad set 4mm buccal to the deciduous molars with metallic anterior bite block. The vestibular components were attached using a 1.0 mm labial wire running adjacent to the dentition, and a 1.1 mm diameter wire crossing the occlusal table and ending in the palatal button. Additional 1.0 mm wires exited each side of the palatal button to form expansion coils extending from the first permanent molars to the deciduous canines (Fig. 2).

The ABPFA had no dental retention and was held in place solely by stimulation of the masticatory muscles. By actively biting on anterior bite blocks, molar extrusion may be induced and vertical growth of the mandibular ramus stimulated. Stimulation from the palatal button may train the tongue to reach its physiologic position near the upper incisors. Maxillary expansion may be induced by the expansion coils, and by the vestibular buttons, which relieve pressure generated by muscles on the dentition. The sagittal position of maxillary incisors was maintained by the vestibular arch.

All patients were instructed to wear the ABPFA for 16 hours per day, i.e. during the night and in the afternoon, and to remove it during eating and brushing. Active treatment lasted 24 months for all patients.

-Statistical analysis

When dealing with the evaluation of effects of functional orthodontic appliances in growing patients, one of the major issues is to isolate modifications of craniofacial structures induced by normal growth from the therapeutic effects of appliances (i.e. modifications of craniofacial structures attributable to the appliances). This can be accomplished only by comparing data of treated patients with a matched untreated control group, which is often very difficult to make, due to the obvious ethical implications of leaving patient without treatment.

Nonetheless, a recent report by Yoon SS et al. has described craniofacial growth of untreated class II patients from ages 9 to 18 years 12. We have used data provided in such a report for comparison with our data. In particular, having hypothesized a non normal distribution of data, a Student’s t-test with two tails and a significance level of 0.05 was used to assess differences of means and standard deviations of our T0 data with Yoon’s series of untreated patients at the age of 9 years. The same statistical approach was then used to assess differences of our T1 data with Yoon’s series of untreated patients at the age of 14 years.

## Results

Angular and linear cephalometric measurements (means and standard deviations) at baseline (T0) and 24 months treatment (T1) are shown in  Table 2 (skeletal modifications) and  Table 3 (dental modifications). In the same tables data of the corresponding measurements from the reference paper 11 have been reported, together with p-values for their comparisons.

**Table 2:**
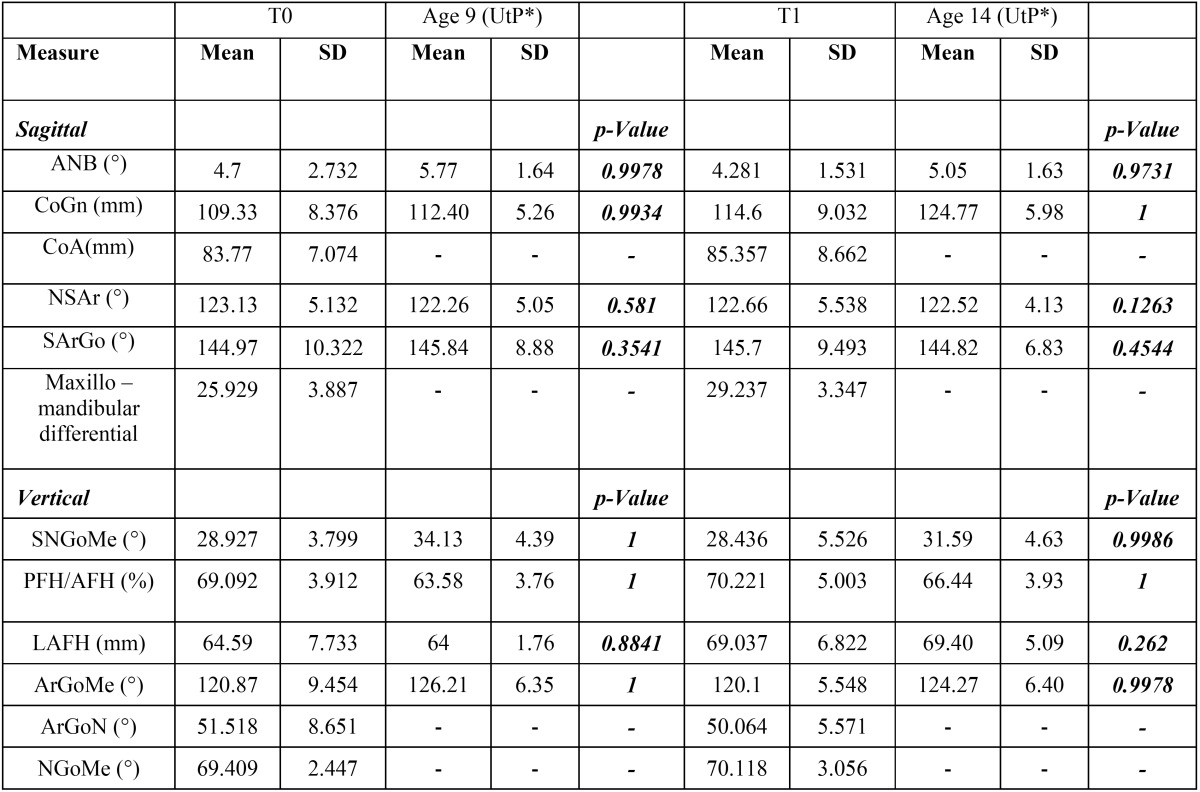
Descriptive statistics and statistical comparisons for cephalometric skeletal measurements. *UtP: untreated patients, data from Yoon SS *et al.* 2015.

**Table 3:**
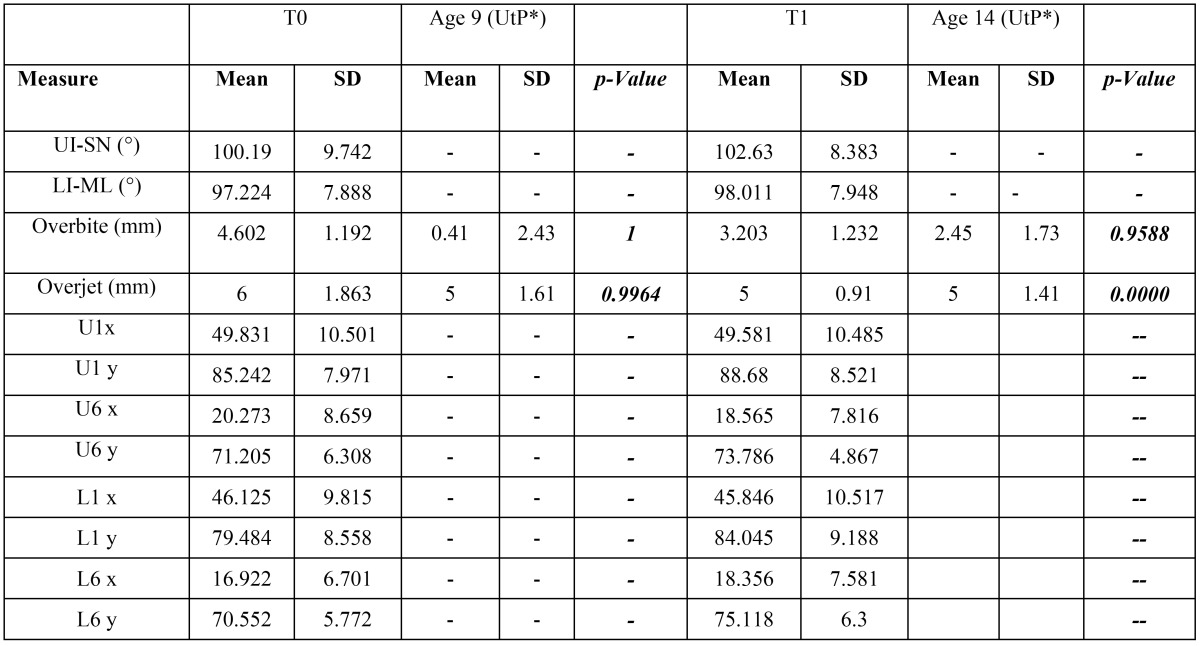
Descriptive statistics and statistical comparisons for cephalometric dental measurements.*UtP: untreated patients, data from Yoon SS *et al.* 2015.

Comparisons made of our T0 data with data of untreated 9 years old patients from Yoon SS *et al.* showed no statistical significant differences, which is a good indicator that our series is very similar and comparable, for the considered parameters, with the reference series. On the other hand, our T1 data, when compared with data of untreated 14 years old patients from Yoon SS et al. showed no statistical significant differences for skeletal parameters, whereas dental parameters showed a significant different overjet, which was significantly reduced (6 mm at T0 vs. 5 mm at T1) in our series.

## Discussion

Class II malocclusion is commonly encountered in orthodontic practices (13). Many aspects related to its physiopathology are extremely relevant for its treatment; first of all, the mandibular growth and its antero-posterior position. Under this point of view, McNamara observed that the mandibular retrusion is a common finding in Class II malocclusion patients (14) and, Stahl et al. reported that from ages 10 to 15, the class II subjects had a significantly smaller increments in mandibular growth compared to Class I patients. A growth deficiency of mandibular ramus height causing backward rotation of the mandible has been also described (15).

On the other hand, it has been reported that the growth in mandibular length was similar between the Class I and Class II patients (16). Another key aspect of Class II malocclusion is the mandibular direction of growth. Siriwat and Jaraback suggested that the Class II was the dominant malocclusion among subjects with hypo-divergent facial growth pattern, i.e. increase of the facial height ratio (posterior facial height/anterior facial height relation) with an increase of horizontal face growth and a reduction of the lower anterior facial height (17).

Functional appliances aiming at treat Class II deep bite malocclusion are strictly related to the possibility to modify both growth potential and direction of the jaws. Nonetheless, orthodontic interventions to correct such a malocclusion with functional appliance remain a controversial issue (18). In the last twenty years, several different appliances have been proposed, but results have been inconclusive or contradictory (19). The most evaluated treatment strategies are: the headgear (HG) (20), bionator (21), the twin block functional appliance (22), Frankel II (23), Herbst appliance (24), Forsus appliance (25), fixed appliance in conjunction with anterior bite plane 6, a fixed anterior bite plane (26) and in severe cases surgery to the jaws in combination with orthodontics may be required (27).

The aim of the functional of fixed/functional treatment is to enhance mandibular growth, improve antero-posterior apical base relationship, and promote favourable dental changes (overbite and overjet correction) and soft tissue modifications (28).

The common effects of the above mentioned treatment strategies are: reduction of overjet (29), increase of pro inclination of the lower incisors and a slight retro inclination of upper incisor (30), a slight maxillary growth restrain (31), mandibular length increase (32) and increase of lower facial height (33).

In the present paper effects of ABPFA in Class II deep bite malocclusion patients was evaluated. This type of functional appliance was built with no forced mandibular advancement and an anterior bite plane; this, may enhance the vertical growth of the mandible and reduce the extrusion of the anterior teeth, as well as removing the “anterior wall” that inhibits the sagittal displacement of the mandible. In addition, the two palatal arms, together with the vestibular buttons, may stimulate maxillary expansion. Our findings showed that the ABPFA had no significant effects on skeletal cephalometric measurements. In fact, although some variations in such measurements were observed, differences were not statistically significant when compared with untreated patients; thus, it seems that such an appliance is not capable to significantly affect the amount of facial structures’ growth. Nonetheless, it has been observed a significant dental effect; in particular, it was found a significant reduction of the overjet and the tendency to a reduction of the overbite. This seems to suggest a significant beneficial effect in mandible displacement by reducing the counter clockwise rotation of the mandible, which is further confirmed by the almost absence of modifications of ArGoMe and SNGoMe angles. Upper and lower incisors inclination was almost unmodified, thus, confirming that the ABPFA is particularly suitable to reduce the non-desirable dental effects of the functional therapy represented by lower incisors pro inclination (34), and upper incisors retro-inclination (35). In fact, after treatment, we observed almost no pro-inclination of the lower incisors (LI-ML angle +0,787°), and a very limited retro-inclination of upper incisors (UI-SN angle + 2.4°) that was much less than that reported for other appliances (- 9.26° with Head Gear, - 4.48° with Bionator, -9.2° with Twin Block, -4.5° with Frankel) (36). The latter, is an extremely significant effect because, by limiting the “anterior wall” of the upper incisors, may facilitate and enhance the mandibular anterior displacement.

## Conclusions

Based on this study’s results, the following conclusions can be made:

1. In ABPFA group, the treatment effects were reduce mainly Class II malocclusion, overjet and overbite alteration.

2. This appliance seems to suggest a significant beneficial effect in mandible displacement by reducing the counter clockwise rotation of the mandible, which is further confirmed by the almost absence of modifications of ArGoMe and SNGoMe angles.

3. The ABPFA is particularly suitable to reduce the non-desirable dental effects represented by lower incisors pro inclination, and upper incisors retro-inclination.
